# Easy Diagnosis of Invasive Pneumococcal Disease

**DOI:** 10.3201/eid1706.100997

**Published:** 2011-06

**Authors:** Laura Selva, Xavier Krauel, Roman Pallares, Carmen Muñoz-Almagro

**Affiliations:** Author affiliations: University Hospital Sant Joan de Déu, Barcelona, Spain (L. Selva, X. Krauel, C. Muñoz-Almagro);; Saint John of God Hospital, Lunsar, Sierra Leone (X. Krauel);; Bellvitge Hospital, Barcelona (R. Pallares);; University of Barcelona, Barcelona (R. Pallares)

**Keywords:** Streptococcus pneumonia, serotypes, dried spot, real-time PCR, pneumococcal disease, bacteria, letter

**To the Editor:** Invasive pneumococcal disease (IPD) causes many cases of severe disease and death among children <5 years of age, mostly in developing countries (*1*,*2*). Before conjugate vaccines can be introduced in developing countries, information about disease epidemiology is urgently needed. The lack of laboratories equipped to perform pneumococcal serotyping leads to the need to send isolates to reference laboratories. Good sample preservation is necessary to prevent samples from arriving at the laboratory in poor condition. We evaluated the usefulness of multiplex real-time PCR from strains and blood samples kept at room temperature on dried blood spot (DBS) filter paper for detecting and serotyping *Streptococcus pneumoniae*. DBS screening is a reliable method that requires only a small amount of blood; it is used for the diagnosis of several human diseases (*3*,*4*).

To validate the technique, we selected 15 pneumococcus clinical isolates representing 15 serotypes (1, 5, 19A, 19F, 14, 3, 7F, 4, 6A, 6B, 8, 9N, 18C, 23A, 23F) obtained during 2009 from patients at Hospital Sant Joan de Déu, in Barcelona. These isolates, used as controls, had been serotyped by quellung reaction at the Instituto de Salud Carlos III, Majadahonda-Madrid, Spain. These strains were cultured overnight at 35°C in 5% carbon dioxide on Columbia agar plates with 5% sheep blood (bioMérieux SA, Marcy l’Etoile, France). A suspension of each strain was adjusted to match a 0.5 McFarland standard (equivalent to 10^8^ colony-forming units (CFU)/mL). Stock solutions of pneumococcus culture for each previously identified serotype were injected into blood previously extracted from 2 healthy volunteers. Serial dilutions of 100,000 CFU/mL to 1,000 CFU/mL (1,000 to 10 CFU equivalents/PCR) were performed. A total of 100 µL of blood was applied to DBS filter paper, and another 100 µL was used for DNA extraction from fresh blood. All DBS samples were air dried for 1 week.

The procedure was also performed on negative control blood samples.

DNA was extracted from DBS and fresh blood samples by using the NucliSense easyMAG automated extraction platform (bioMérieux, Boxtel, the Netherlands) according to the manufacturer’s instructions. DNA detection of the *pneumolysin* (*ply*) gene by real-time PCR was performed according to a published assay (*5*). In addition, we performed a multiplex real-time PCR for molecular serotype detection of serotypes 1, 3, 5, 4, 6A, 6B, 7FA, 8, 9VANL, 14, 15BC, 18CB, 19A, 19FBC, 23F, 23A and the conserved capsular gene *wzg* as described by Tarrago et al. (*6*). DNA extracts were amplified with the Applied Biosystems 7300 Real-time PCR System (Applied Biosystems, Foster City, CA, USA). Negative results were defined as those with cycle threshold >40.

To evaluate the reliability obtained with this in vivo approach, we performed identification and serotyping of *S. pneumoniae* in 25 DBS samples from 25 children at Saint John of God Hospital in Mabesseneh-Lunsar, Sierra Leone. This hospital does not perform blood cultures. IPD was confirmed when DNA of a *pneumolysin (ply)* gene and an additional capsular gene of *S. pneumoniae* were detected by multiplex real-time PCR of DBS samples.

Detection of *ply*, *wzg*, and the specific gene for molecular serotype showed that both fresh blood and DBS samples yielded correctly positive results from the 10-fold serial dilutions analyzed (Table). With respect to the 25 (11 female and 14 male) patients from Sierra Leone who had suspected IPD, the median age was 25.71 months (range 15 days to 96 months); all had a diagnosis of fever without apparent source, and 16 also had malaria. Of these 25 children, DBS samples from 15 (60%) yielded a positive result for the *ply* and *wzg* genes, so they were considered confirmed episodes of IPD. A serotype included in 13-valent conjugate vaccine was detected in 6 (40%) of 15 positive samples: serotypes 3, 7FA, 19A, 6A, 6B, and 9VNL (1 sample each). In the remaining 9 samples, the results for *ply* gene and *wzg* gene were positive, but none of the 24 tested serotypes was detected.

**Table:** Sensitivity of real-time PCR for detecting *Streptococcus pneumoniae* *ply* or *wzg* genes or a specific gene for molecular serotype from fresh or dried blood spot samples*

Gene or serotype	Serial dilutions correctly detected, CFU equivalent/PCR†	
	Fresh blood	Dried blood spot
*ply* gene	1.10^1^–1.10^3^	1.10^1^–1.10^3^
*wzg* gene	1.10^1^–1.10^3^	1.10^1^–1.10^3^
Serotype 1	1.10^1^–1.10^3^	1.10^1^–1.10^3^
Serotype 5	1.10^1^–1.10^3^	1.10^1^–1.10^3^
Serotype 19A	1.10^1^–1.10^3^	1.10^1^–1.10^3^
Serotype 19F	1.10^1^–1.10^3^	1.10^1^–1.10^3^
Serotype 14	1.10^1^–1.10^3^	1.10^1^–1.10^3^
Serotype 3	**1.10^2^–1.10^3^**	1.10^1^–1.10^3^
Serotype 7F	1.10^1^–1.10^3^	1.10^1^–1.10^3^
Serotype 4	1.10^1^–1.10^3^	1.10^1^–1.10^3^
Serotype 6A	1.10^1^–1.10^3^	**1.10^2^–1.10^3^**
Serotype 6B	1.10^1^–1.10^3^	1.10^1^–1.10^3^
Serotype 8	1.10^1^–1.10^3^	1.10^2^–1.10^3^
Serotype 9N	1.10^1^–1.10^3^	1.10^1^–1.10^3^
Serotype 18C	1.10^1^–1.10^3^	1.10^1^–1.10^3^
Serotype 23A	1.10^1^–1.10^3^	1.10^1^–1.10^3^
Serotype 23F	1.10^1^–1.10^3^	1.10^1^–1.10^3^

*Serial dilutions (10–1,000 CFU equivalent/PCR) of 15 pneumococcus cultures mixed with 15 negative-control blood samples were analyzed. **Boldface** indicates results that differ from others. †10 CFU equivalent/PCR = 1,000 CFU equivalents/mL blood.

This preliminary study enabled us to demonstrate that DBS screening is a reliable and easy method for diagnosing IPD and also for epidemiologic surveillance of the more frequent serotypes. The main limitation of our study is the small number of DBS samples sent from Saint John of God Hospital in Sierra Leone.

In conclusion, the DBS technique enables reproducible transport of samples for identification and serotyping of *S. pneumoniae* by multiplex PCR. The use of DBS on filter paper is an attractive alternative method for storing samples at room temperature and easily transporting them. Additional studies, including evaluation of the relative sensitivity of this method compared to direct culture, are necessary.
